# Application of near infra-red laser light increases current threshold in optic nerve consistent with increased Na^+^-dependent transport

**DOI:** 10.1007/s00424-024-02932-1

**Published:** 2024-02-29

**Authors:** Hin Heng Lo, Tawan Munkongcharoen, Rosa M. Muijen, Ritika Gurung, Anjali G. Umredkar, Mark D. Baker

**Affiliations:** Neuroscience, Surgery and Trauma, Blizard Institute, QMUL, Whitechapel, London, E1 2AT UK

**Keywords:** Optic nerve, Current threshold, Infra-red laser, Membrane excitability

## Abstract

Increases in the current threshold occur in optic nerve axons with the application of infra-red laser light, whose mechanism is only partly understood. In isolated rat optic nerve, laser light was applied near the site of electrical stimulation, via a flexible fibre optic. Paired applications of light produced increases in threshold that were reduced on the second application, the response recovering with increasing delays, with a time constant of 24 s. 3-min duration single applications of laser light gave rise to a rapid increase in threshold followed by a fade, whose time-constant was between 40 and 50 s. After-effects were sometimes apparent following the light application, where the resting threshold was reduced. The increase in threshold was partially blocked by 38.6 mM Li^+^ in combination with 5 $\mu$M bumetanide, a manoeuvre increasing refractoriness and consistent with axonal depolarization. Assessing the effect of laser light on the nerve input resistance ruled out a previously suggested fall in myelin resistance as contributing to threshold changes. These data appear consistent with an axonal membrane potential that partly relies on temperature-dependent electroneutral Na^+^ influx, and where fade in the response to the laser may be caused by a gradually diminishing Na^+^ pump–induced hyperpolarization, in response to falling intracellular [Na^+^].

## Introduction

We have previously reported that the application of near-infra-red (IR) laser light at 1550 nm gives rise to threshold changes in the optic nerve that is probably caused by local warming, through the photo-thermal effect, and an associated hyperpolarization [2]. This was the first description of light reversibly reducing axonal excitability. Such laser light–induced changes in temperature, in small solution volumes (2 ml), without flow, amount to + 2 °C [2]. Alterations in temperature of this magnitude when applied to myelinated axons in the periphery are predicted to cause very small changes in excitability [e.g. 13, 20]. Cooling peripheral nerve has effects on Na^+^ channel gating kinetics that affect threshold and refractoriness (i.e. the channels respond to a stimulus current more sluggishly) [13]. Raising the temperature of peripheral axons from near 32 to 40 °C is expected to cause a hyperpolarization of only − 1–2 mV and increase resting potassium conductance [20]. In contrast, we now associate our laser light illumination with changes in threshold of around 10% for the optic nerve (consistent with substantially larger hyperpolarizations), and we already know a fall in potassium conductance occurs with warming these axons [2, 14]. In light of these findings, a functional difference between the optic nerve and peripheral nerve fibres may be being revealed. Finally, when comparing the effects of temperature on recovery cycles in optic nerve and human sensory nerve fibres, the optic nerve fibres appear far more sensitive to temperature swings between 37 °C and room [22].

In this manuscript we have not been able to measure the temperature changes directly in the nerve (as the nerve is small, and only 1 mm in diameter); however, we have been able to measure functional changes in the illuminated axons in response to laser light application. That the laser light induces a photo-thermal effect is consistent with the mechanism of noxious thermosensation in skin measured using the Hargreaves’ test e.g. [29]. We have now taken our observations on the effects of IR laser light application further in order to glean more information about the mechanisms involved, and this forms the overall objective of the study. A more detailed description of the objectives is given below. We have been able to measure the power output of the laser diode using a 400 pA continuous driving current, as 10 mW [2], and so the experimental set-up has some similarities to the application of near IR laser light through a microscope objective to study the dynamics of cell membrane incorporation or vesicle recycling [3].

We have made a case that local warming hyperpolarizes the axons. Evidence in favour of this includes the direct extracellular measurement of membrane potential hyperpolarization (demarcation potential), during illumination, and the finding that laser light application increased the amplitudes of depolarizing after-potentials that follow an evoked action potential [2]. Circumscribed warming by the light application at the stimulating electrode consistently increased the threshold, and the threshold increase was made larger by blocking *I*_h_ [2]. We have previously associated warming the whole optic nerve with a hyperpolarization [2, 14] and provided evidence that the electrogenic Na^+^-pump probably mediates this, an action reasoned to be dependent upon the influx of Na^+^ by a mechanism predicted to be electroneutral [1]. Such an influx might be provided by solute transport involving either a substrate or substrates and or Cl^−^ ions. Both the influx and efflux of Na^+^ ions would be expected to be temperature-dependent, and the temperature dependence of influx appears consistent with a *Q*_10_ of 3 [2]. In order to more formally describe the effects of warming, we have introduced a term *I*_EN_ (pump-current resulting from electroneutral Na^+^ entry) in an equation that allows the calculation of axonal membrane potential (Austerschmidt et al. [2], Eq. 3). This approach can be thought of as a hypothesized transmembrane Na^+^ ion cycle, where warming is expected to hyperpolarize the membrane potential and decrease excitability.

The objectives of this study were to provide more information about the dynamics of threshold changes brought about by IR laser light application in optic nerve axons, including by applying the light for several minutes and also by using two sequential illuminations with a variable delay. We have explored a resulting axonal depolarization and a diminution of the effects of the laser, without a change in the ambient temperature, following attempts to reduce Na^+^ entry. In this manuscript, we have attempted to determine if there is any evidence that the addition of a putative substrate for Na^+^-dependent transport can alter these dynamics. Our previous work was unable to rule out the possibility that IR laser light altered the properties of myelin (and this is important because a fall in trans-myelin resistance would be expected to impact excitability without the necessity of changing the membrane potential). Therefore, one of the objectives of the present work was to discover whether there is any evidence for an effect on myelin, by using a measure of trans-myelin resistance called fast electrotonus. Finally, we have demonstrated that our previous theoretical approach to understanding the effects of gross temperature changes on nerve [1, 2] can reasonably predict the rapid changes brought about by the laser light, but not the fade of threshold increase. The speculated mechanism of warming-induced hyperpolarization is dependent on Na^+^ movement on transport mechanisms, although direct measurements of intra-axonal Na^+^ ion concentrations, that may support this hypothesis, are currently unavailable.

## Methods

### Animals

Adult male and female wild-type Wistar rats and female wild-type Sprague Dawley rats (~ 320 g) were obtained from Charles River, Manston, Kent, and accommodated under veterinary supervised conditions, in a Home Office–designated establishment (Barts and the London QMUL), with food and water ad libitum. The animals underwent euthanasia by exposure to a rising concentration of CO_2_ and subsequent cervical dislocation, as specified by the guidance in the Schedule 1 protocol list and in accordance with UK legislation (no further ethical approval is required under UK law), published by the Home Office UK (Scientific Procedures Act 1986). The animals were culled by a qualified researcher under the Schedule 1 licence of the establishment. Optic nerves were removed following euthanasia.

### Recording

**Fig. 1:**
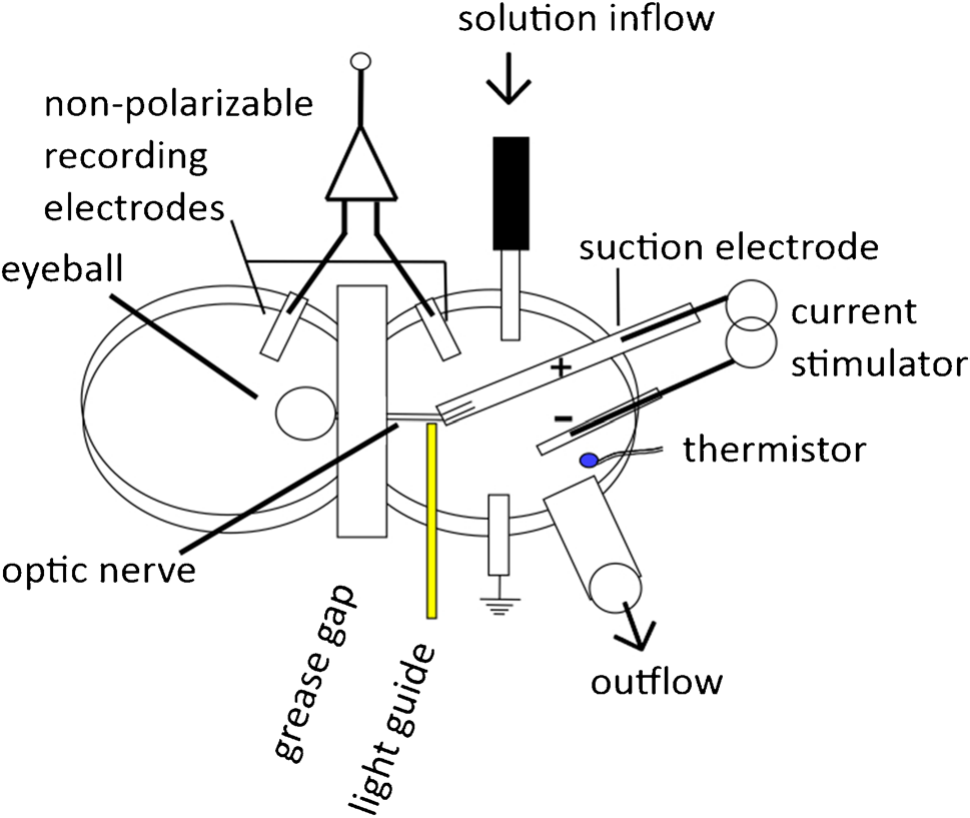
The nerve bath comprised two chambers, both filled initially with control buffer solution. The chambers were separated by a grease gap, through which the optic nerve was mounted. Recordings of the action potential were made using non-polarizable electrodes positioned in both chambers, allowing differential recording across the gap. Stimulating and earth electrodes were present in the right-hand chamber (as shown here). The right-hand chamber also allowed for continuous solution flow, from a heated input, and the temperature was monitored using a thermistor. The degree of heating was continuously adjusted to allow for a constant gross temperature in the right-hand chamber. A laser light guide, touching the optic nerve, brought about highly localized warming at the site of stimulation. The nerve bath was continuously supplied with O_2_ using a bubbler (not shown), and drugs and experimental solutions were introduced into the right-hand chamber. Perfusion temperature was 32–36 °C

Details of the custom-made recording bath and the process for mounting the optic nerve have appeared previously, e.g. Austerschmidt et al. [1, 2]. Briefly, the eyeballs and attached optic nerves were isolated into the oxygenated buffer, cleaned of adherent tissue, and subsequently mounted into a two-chambered nerve bath (Fig. 1). When in use, the chambers of the bath were functionally separated by a barrier that was filled with Vaseline after the nerve had been threaded across. For ease of loading the nerve through the gap between the chambers, the nerve was sucked inside a plastic venous cannula (outer diameter 1 mm) that was able just to fit the gap, and that had an internal diameter only just larger than the diameter of the nerve. Once the nerve was pulled into the right-hand chamber (see Fig. 1), this cannula was subsequently removed, and the chiasm end of the nerve was picked up by a suction electrode.

The right-hand chamber was constantly provided with an oxygenated buffer solution at a known, near physiological temperature. Perfusion temperature was always maintained between 32 and 36 °C, and more detail is given in the figure legends. The chiasm end of the optic nerve was picked up by a plastic suction electrode, as previously described, formed from a venous cannula [2] (that allowed for electrical stimulation) so that evoked action potentials initiating at the end of the electrode travelled retrogradely towards the grease barrier. This solution flow also accommodated the control solution and also solutions with altered ionic composition and bumetanide, accessing the site of stimulation at the end of the suction electrode that was also the site of laser light application. The left-hand chamber contained the eyeball, still in continuity with the nerve. Differential recordings of action potentials were made across the grease-gap at the barrier, using non-polarisable recording electrodes on both sides and signal amplification using a high-impedance differential amplifier (Warner instruments DP-311, with band-pass settings for action potential recording, between 10 Hz and 3 or 10 kHz), Fig. 1.

The nerve was stimulated using 200 µs constant-current pulses generated by a computer-controlled stimulator (Digitimer DS4, Welwyn Garden City, Herts, UK) that provided a continuous amplitude modulation. Stimulus current amplitude was controlled by the data collection programme QTRAC-S (Hugh Bostock, Institute of Neurology, UCL, Queen Square, London; available through Digitimer. https://www.digitimer.com/product/life-science-research/software/qtracw-threshold-tracking-software/). The stimulus amplitude was maintained as close to 5% of the supramaximal response as allowed by the threshold-tracking software, with a 10% amplitude error margin within the response window, and this corresponded to an action potential attributable to what we have called the F-fibres in the nerve (the axons with the largest diameter and the highest conduction velocity, that give rise to the earliest component of a polyphasic action potential response to large currents), corresponding to the axons recruited in response to the smallest, brief duration stimulus currents, e.g. [2, 15, 22, 33]

### Recovery cycles

Recording a recovery cycle required two electrical stimuli, one providing a conditioning response and one a test response, and this stimulus protocol was accommodated using a second channel in QTRAC-S. Such a protocol allowed the estimation of the stereotypical changes in threshold that follow an action potential. The conditioning stimulus in this recording channel was applied between 3 and 500 ms before the test stimulus. During the recording of a recovery cycle, the delay between conditioning and test stimuli (the inter-stimulus interval, ISI) was incrementally increased, once the response error requirement (in this case 10%, see above) was met for the target response with the current ISI. This means that the programme hunted for the threshold and only progressed through the cycle when the threshold criterion was met for any previously chosen ISI. A normal recovery cycle for a myelinated axon includes a period of relative refractoriness, with a short ISI, followed by a period of superexcitability at longer ISIs, and this is exemplified by optic nerve recordings.

### Electrotonus

In order to investigate changes in myelin resistance potentially caused by laser light application, electrotonus was recorded. During recordings of electrotonus, sub-threshold polarizing currents that could be either 0.2 times the current threshold-current (depolarizing) or − 0.4 times the current threshold-current (hyperpolarizing) were applied at the site of stimulation. Where the length of the nerve in the right-hand chamber was short, it was possible to record the evoked changes in membrane potential in the nerve fibres across the barrier (Fig. 6) and also to record the resulting changes in the threshold at the site of stimulation. The significance of the short length of the nerve in the right-hand chamber (Fig. 1) is to allow the measurement of potential and threshold changes at sites that are close together. The fast phase of electrotonus, i.e. the change in potential largely dependent on the resistance across the myelin, was sampled on each cycle of QTRAC-S, allowing us to follow any changes in resistance.

### Laser light irradiation

The laser diode generated near-IR light at 1550 nm (PL15D005100A-0–0-01; Laser Components (UK) Ltd, Chelmsford, Essex, UK). It was driven from a constant-current bench power supply (Thurlby-Thandar id, Huntingdon, Cambs UK), using continuous currents of 400 mA, only. The light application was therefore continuous, not pulsed. The laser diode used had a flexible integral fibre optic (9 µm diameter) that could be made to illuminate locations along the optic nerve. It was stably supported by a micromanipulator and in these experiments was used to locally warm only close to the site of stimulation, at the end of the stimulating electrode. The contact between the end of the fibre optic and the nerve appeared to be critical, as the observed changes in threshold were found always to occur only when we had reason to believe physical contact was made (giving rise to a slight lateral displacement of the nerve), under the perfusion solution of the bath right-hand chamber. Experiments estimating energy output and measuring temperature changes at the end of the light guide previously reported [2] are consistent with temperature changes of + 2 to 3 °C in the tissue.

### Solutions and drugs

The control buffer solution is composed of the following: NaCl 140, HEPES hemi Na 10, CaCl_2_ 2.1, MgCl_2_ 2.12, KCl 2.5, glucose 10, with concentrations in mM. For solutions with Li^+^, incorporating reduced Na^+^, the solution contained the following: NaCl 101.4, LiCl 38.6, HEPES hemi Na 10, CaCl_2_ 2.1, MgCl_2_ 2.12, KCl 2.5, glucose 10, with concentrations in mM. Bumetanide was obtained from Tocris (Bio-techne Ltd, Abingdon, Oxfordshire, UK) and made up as a stock solution in DMSO, at 5 mM and stored at − 20 °C. Bumetanide is a loop diuretic that blocks anion-cation cotransport, e.g. [18]. The concentration of vehicle in the nerve bath never exceeded 0.1%. All perfusing solutions were adjusted to pH 7.2–7.3 with the addition of small quantities of HCl, when required. Salts and buffers were obtained from Merck Life Science Ltd (Gillingham, Dorset, UK).

### Statistical analysis

Statistical comparisons were carried out using paired *t*-tests, with the critical value of *P* taken as 0.05. Best-fit, non-linear regression was used for curve fitting. Experimental values are plotted or quoted as means ± SEM wherever possible. Statistical analysis was carried out in Microsoft Excel.

## Results

### Paired light application reveals a suppression and a slow recovery of response to illumination

**Fig. 2:**
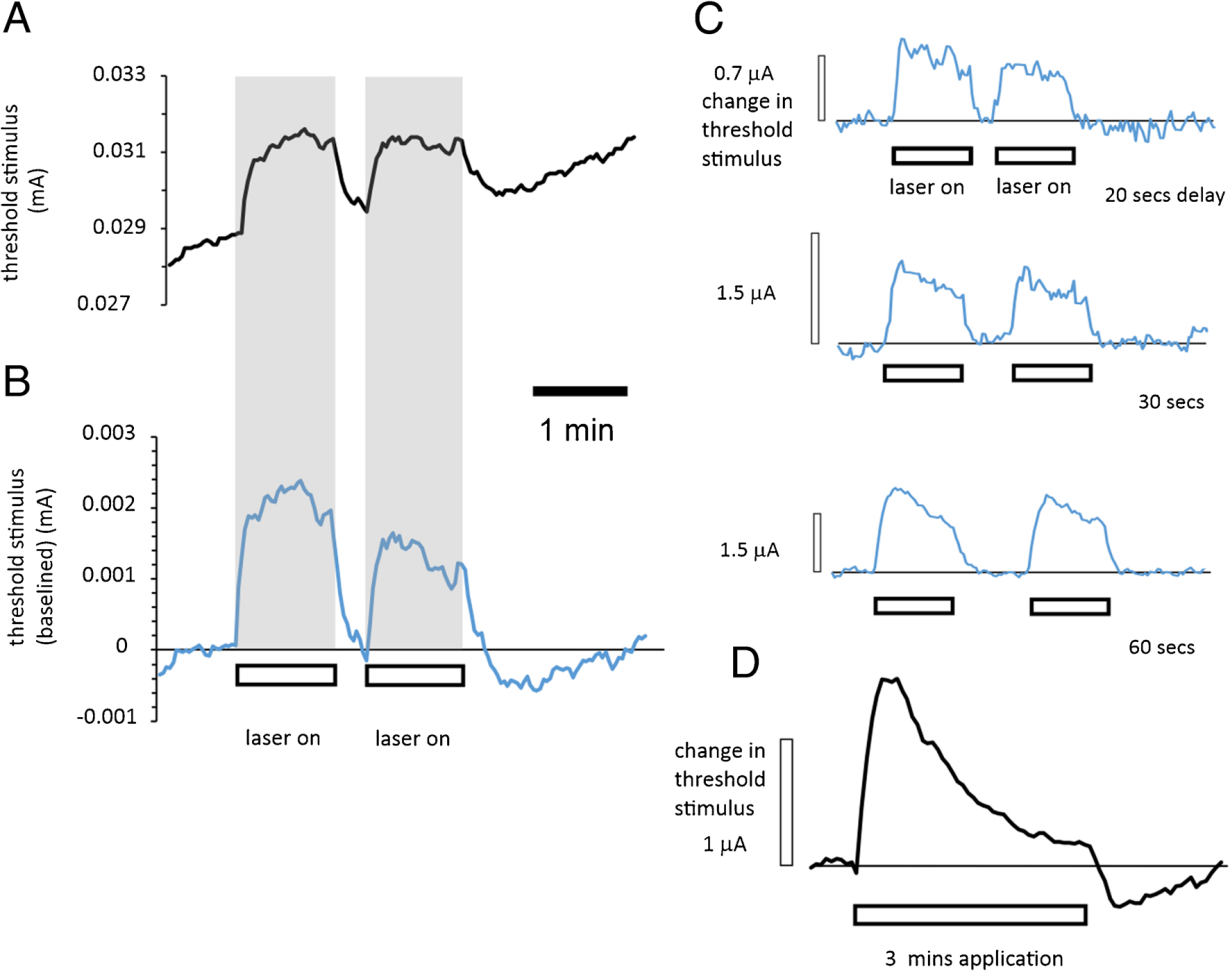
Application of near-IR laser light at the site of stimulation reliably increased the threshold of optic nerve axons and paired applications diminished the peak change in threshold. **A** Raw threshold-tracking trace in QTRAC, before baseline correction, showing the threshold changes elicited by applying a pair of laser light illuminations for 1 min. **B** Same data following linear baseline correction. As the delay in applying the second light application is brief (20 s), the peak change in threshold is smaller. Following the second light application, there is evidence for an apparent after-effect, where the threshold is transiently reduced. **C** Introducing longer delays between paired laser light applications reveals that the peak change in threshold slowly recovers. **D** Application of a single laser light illumination for 3 min reveals the threshold increase can fade almost completely and is also apparently associated with an after-effect with a reduced threshold. Perfusion temperature was 33–35 °C

The laser-induced effect on the membrane potential must be rapid, because the threshold increase in illumination is almost immediate, as can be seen in Fig. 2. This suggests that the Na^+^ pump activity in the axons responds quickly.

**Fig. 3:**
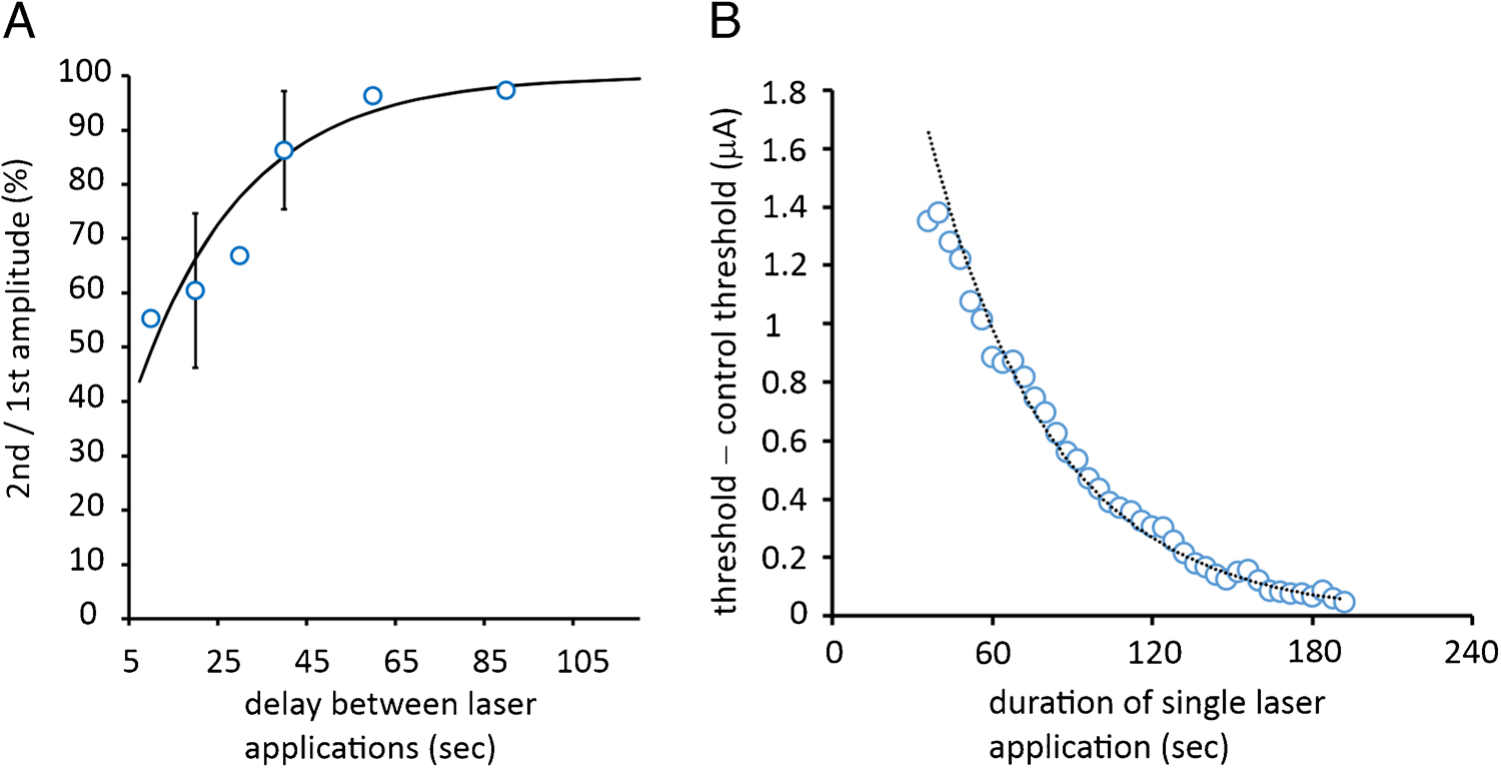
Recovery of suppression of peak threshold change, seen on the second of a pair of light applications, has a best-fit time constant of 24 s while the fade with a single light application for 3 min has a longer time constant. **A** Best-fit exponential, *R*^2^ = 0.918, $\tau$ = 24.4 s, *n* = 5. These data reveal the dynamics in axonal response to laser light where complete recovery from a 1-min application requires close to 2 min. **B** Time-constant associated with fade in threshold increase caused by a single laser light application is longer at 46.1 s, *R*^2^ = 0.984 (*n* = 3 recordings from two nerves gave us a time constant of between 40 and 50 s), but neither the onset of threshold fade nor the recovery of the threshold increase is likely to be explained by intra-axonal Na^+^ ion diffusion alone, because they are too slow. Perfusion temperature is 33–35 °C

Applying laser light for 1 min durations at the site of stimulation gave rise to consistent increases in the threshold, where the peak can be measured quite easily, if precautions are taken to eliminate small changes in baseline threshold that occur over a few minutes of continuous recording. We followed the first period of light application by a second, again for 1 min, with a delay that varied between 20 and 90 s (with a much longer cycle repeat of over 2 min). Expressing the difference in peak threshold increase in response to the second illumination, as a percentage of the first, allowed us to plot the recovery of the peak where the best-fit time constant to the average values is 24 s (Fig. 3A). This result implies that complete recovery from a single 1-min application of light requires up to 2 min. One possibility is that this recovery of the maximal threshold change represents the recovery of the local intracellular Na^+^ concentration, depleted during light application by increased activation of the Na^+^ pump. If this replenishment was caused only by diffusion of Na^+^ ions already within the axoplasm, then the time-constant might have been expected to be shorter than this (see discussion), suggesting that this may be the time constant associated with gross concentration changes of intracellular Na^+^, caused by differences in the effect of the laser on Na^+^ influx and efflux. In contrast, the fade in threshold increase during a single, long-lasting laser light application we hypothesize is caused by ATPase-driven depletion of intra-axonal Na^+^ that gradually causes a decline in the pump-current *I*_EN_ through a mismatch between Na^+^ influx and efflux.

### Long-lasting laser light applications reveal a fade in the response to illumination

We used laser light applications of 3 min to explore the time constant associated with the fade in threshold increase and discovered that the best-fit exponential decline gave us a time constant between 40 and 50 s (Fig. 3B), although we have seen a partial slow decay in threshold-increase in most nerves studied with 1-min duration illuminations or longer. During some briefer laser light applications, the resulting increase in threshold was not observed to fade, and we suggest this might be a result of a high resting intra-axonal Na^+^ ion concentration before the light was applied and assume that prolonging the exposure would have eventually caused a fade. A possible cause for concern in the interpretation of the threshold changes resulting from long-lasting light applications is that there may have been a fade in the light output from the laser diode over several minutes of continuous operation. But this fear we have been able to partially allay because we have recorded evidence for the after-effects of long-lasting light application (e.g. Figure 2D), consistent with the fade being a physiological phenomenon (rather than an artefact generated by the diode), and measurements of water temperature increases caused by the laser were stable for times longer than 2 min (Austerschmidt et al. [2], their supplementary data).

### Lithium and bumetanide depolarize optic nerve axons and suppress the response to the laser

Previously, Austerschmidt et al. [1] reported that exposure of the nerve to extracellular 38.6 mM Li^+^ (that we have suggested is a putative Na^+^-dependent transport blocker), in combination with 5 $\mu$M bumetanide (NKCC1 blocker and loop diuretic) gave rise to changes in axon behaviour consistent with a depolarization of the axons at near physiological temperatures. This is primarily because the combination gives rise to large increases in refractoriness, very similar to that brought about by cooling [14, 22]. A role for the NKCC1 transporter in optic nerve axons began to be revealed because the functional effects of the drug bumetanide on recovery cycles were indistinguishable from that of removing extracellular Cl^−^ ions [1]. Further, the temperature-dependent collapse of the action potential in ouabain-exposed nerves appeared to be slowed by bumetanide, consistent with NKCC1 bringing Na^+^ ions into the axons [22]. The concentration of Li^+^ chosen was an attempt to generate minor changes in excitability (because Li^+^ is known to go through Na^+^ channels, e.g. [19]), while allowing the possibility of transport inhibition. For example, that of neutral amino acid co-transport with Na^+^ [4] on the previously identified transporter in these axons [17]. SLC38A7 and SLC38A8, the human brain analogues, are Na^+^-coupled glutamine transporters that are expressed in central nervous system neurons, and they do not tolerate Li^+^ [8]. This notion of being intolerant to Li^+^ really means that Li^+^ is a poor substitute for Na^+^ in the transport mechanism.

**Fig. 4:**
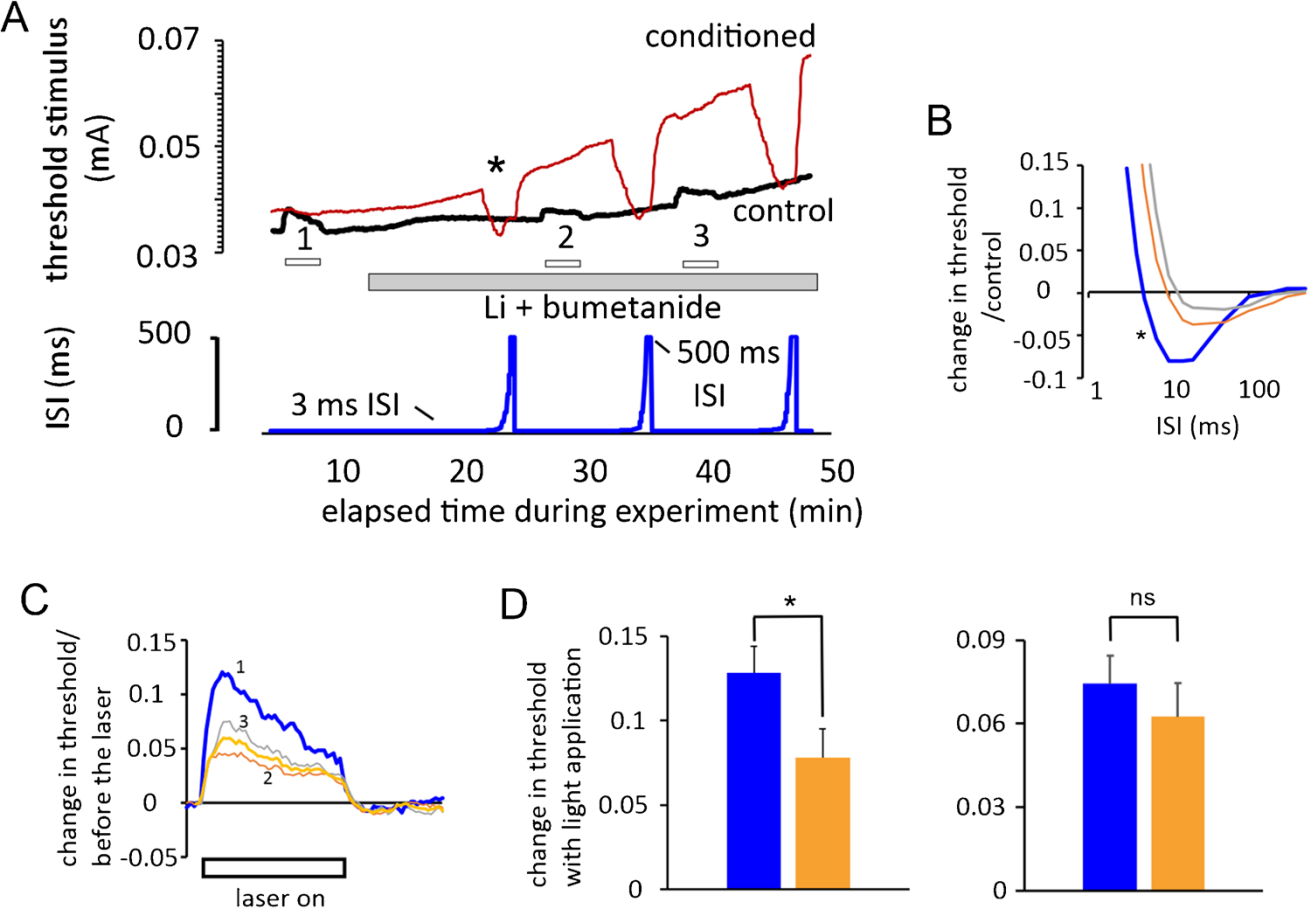
Superfusion of Li^+^ and bumetanide increases refractoriness in optic nerve F-fibres and reduces the threshold changes caused by near IR laser light. **A** Top, continuous threshold measurement in control condition (black trace, control) and following a conditioning action potential (red trace, conditioned). The conditioning stimulus was initially applied with a 3-ms inter-stimulus interval (ISI) seemingly near zero on the ISI plot, but also with an incrementing interval (blue trace, lower panel), revealed in sections of trace going upwards. Li^+^ and bumetanide were perfused into the bath (grey bar), giving rise to an increase in refractoriness following a conditioning stimulus (red trace moves upwards), consistent with membrane potential depolarization, and a reduction in the threshold changes caused by the laser light (sequential applications of laser light, 1, 2, 3). **B** Plot of the recovery cycles generated from the threshold data in **A** (where ISI becomes the *x*-axis), beginning from the asterisk in **A**, in the order blue 1 (asterisk), orange 2, grey 3. The axons are refractory at 3 ms in the recovery cycles shown. **C** Threshold increases evoked by sequentially applied illuminations (1, 2, 3) were reduced by the Li^+^ and bumetanide in the buffer solution, with the threshold increases labelled according to the order of the laser light application in **A**. Mean reduced amplitude is 50% of the pre Li^+^ and bumetanide level (yellow trace), largest response (i.e. 3) at 64% of the pre-exposure response. **D** Left panel: average changes in threshold relative to threshold immediately before light application. Control (blue) 12.8%, and following exposure to Li^+^ and bumetanide (orange) 7.8%, a fall to 60.8% of control (*n* = 6, *P* = 0.016, paired *t*-test. Remains significant following a Bonferroni correction for simultaneous tests). Right panel: In similar experiments without superfusion of Li^+^ and bumetanide and elapsed periods of 20 min between illuminations, there was no significant change in threshold increase (*n* = 4, paired *t*-test). Solution perfusion temperature did not change during these recordings (32–35 °C) and threshold changes shown in **A** are raw data and not corrected for drift over time

The effects of Na^+^ transport inhibition gave rise to changes in the recovery cycle that appeared very similar to those caused by cooling or exposure to raised [K^+^] at a constant temperature [14] and therefore consistent with a depolarization. It was thus important to test whether Li^+^ and bumetanide suppressed the changes in threshold brought about by laser light application. Blocking Na^+^ entry should reduce the effects of the laser, by suppressing the temperature-induced change in *I*_EN_ (from Eq. 3 in Austerschmidt et al. [2]), and this is what happened, Fig. 4. We have already shown that cooling the perfusion solution, from 33 to 26 °C, reduces the response to laser light (Austerschmidt et al. [2], their Fig. 6). Our findings also probably indicate that in the presence of Li^+^ ions and bumetanide, the resting intra-axonal [Na^+^] is depleted, because the refractoriness of the axons progressively increases, consistent with a gradual depolarization and a fall in the resting Na^+^-pump current.

### Effects of potential substrate superfusion on the response to prolonged light application

**Fig. 5:**
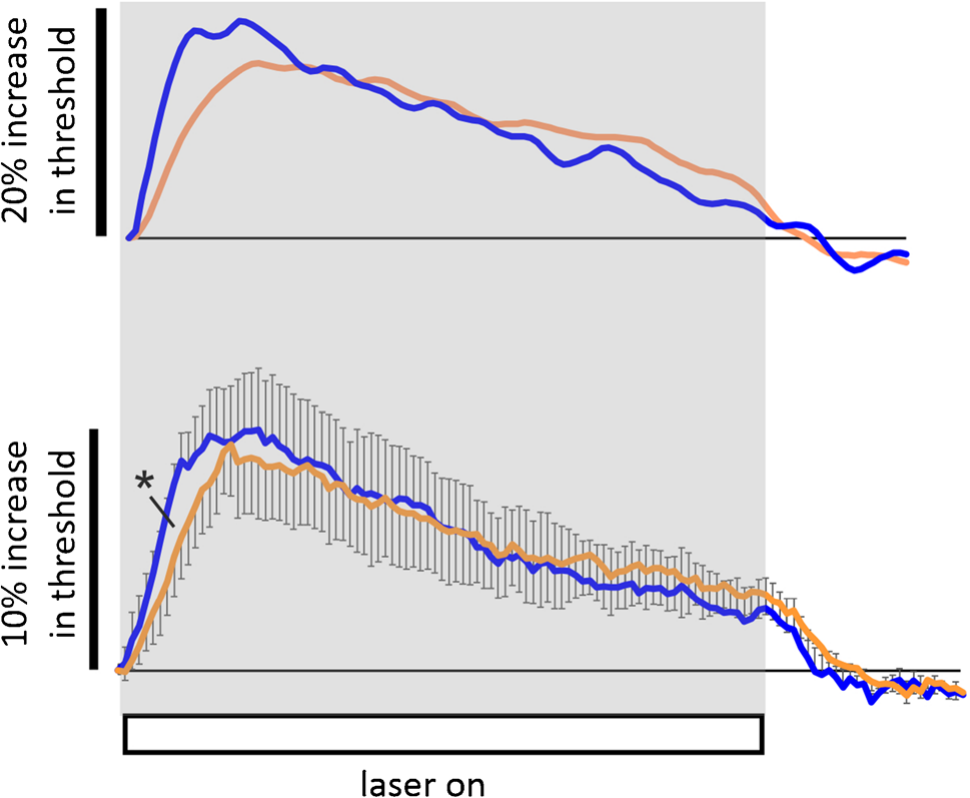
Raising extracellular glutamine to 10 or 20 mM may change the response to the laser, with an apparent drop in the rate of threshold increase and slower fade. Upper panel, for example, average responses from a single nerve, with control buffer solution (blue) and 20 mM glutamine (orange), and at 35 °C. Original threshold-tracking data values smoothed by averaging five adjacent points. The lower panel shows the control responses from four nerves (blue) and responses from the same nerves following both 10 *and* 20 mM glutamine (orange—where both concentrations were studied the data were averaged for individual nerves), means ± SEM, with a significant difference between the two data sets at 20 s into the light application, *P* = 0.046, paired *t*-test. Laser light application during the open bar, for 3 min. Perfusion temperature 33–35 °C

One possible reason for the fade in the threshold increase in response to the laser is a depletion of the substrate transported with Na^+^ across the membrane, although this substrate, or collection of substrates, is presently unknown. Glutamine is importantly transported across the pre-synaptic membrane forming part of the glutamate cycle, to replenish the excitatory neurotransmitter glutamate [4]. Further, it is known that optic nerve axons express a NAT (neutral amino-acid transporter) [17] that is not confined to the pre-synaptic terminal and is normally expected to transport glutamine across the membrane. Finally, it is known that CSF normally contains ambient glutamine at around 500 $\mu$M [16]. We have therefore added glutamine to the buffer solution up to a concentration of 20 mM and can report that this has subtle effects on the threshold response to laser light illumination, Fig. 5. Glutamine may therefore be involved in Na^+^-dependent transmembrane transport, with its depletion in the periaxonal space contributing to the fade in threshold and diminishing Na^+^ influx. The subtle effects include an apparent reduction in fade of threshold increase, a reduction in peak change, and a slowing of the rate of threshold increase at the beginning of the light application (possibly consistent with a raised resting intra-axonal Na^+^ ion concentration caused by minutes of exposure to the amino-acid). The fade in threshold increase may ultimately be explained by a falling intracellular Na^+^ ion concentration, discussed below, and the limited nature of the changes in response to the laser probably means that the aqueous pathway traversed by the glutamine is tortuous.

### Laser light does not change myelin as the axonal input resistance does not change

One possible mechanism by which the application of laser light to the optic nerve could give rise to an increase in electrical excitation threshold is through a reduction in the trans-myelin resistance, and we previously suggested this may be happening, in order to explain fast changes in threshold [2]. Any such change in myelin characteristics would be associated with a fall in excitability because currents applied to the nerve would give rise to smaller depolarizations at nodes of Ranvier, the current being shunted internodally. Thus, it was important to try to determine if myelin was being affected by the applied light, and our results suggest that myelin resistance appears to be unchanged.

**Fig. 6:**
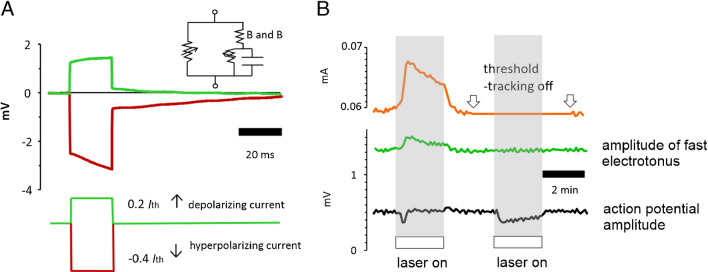
Amplitude of fast electrotonus does not change when laser light is applied. **A** Measuring the fast electrotonic responses to the application of sub-threshold polarizing currents provides a method for continuously monitoring the resistance across the myelin (i.e. B and B, Barrett and Barrett [7]), according to the accepted equivalent circuit of the myelinated nerve. **B** When the laser illuminates the nerve, at the site of stimulation, the rapid fall in the height of the compound action potential (black trace) is quickly compensated by threshold tracking (orange trace). The change in stimulus current necessary to provide the compensation (a little over 10%) is seen in the response of the orange trace. Because the QTRAC tracking programme was set up to keep the polarizing current at a required fraction of the stimulus current, there is also a simultaneous increase in the apparent amplitude of fast electrotonus. However, when the threshold-tracking is subsequently switched off, the amplitude of the action potential remains uncompensated (black trace), clearly falling during illumination, indicating units are lost from the response, and there is no change in the continuously sampled amplitude of the fast electrotonus. This reveals no change in the myelin resistance. Paired *t*-test on hyperpolarizing electrotonus amplitude *P* = 0.116, *n* = 4, ns. Perfusion temperature 32–35 °C

In order to measure a transmyelin resistance, we had to record fast electrotonus, a phenomenon produced by passing either depolarizing or hyperpolarizing currents into the nerve that were not large enough to generate action potentials, but sufficiently large to produce changes in potential that were easily recorded across the barrier in the nerve bath. Fast electrotonus therefore represents changes in electrical potential across the nodes of Ranvier and myelin sheath in parallel, as explained by the Barrett and Barrett model of the nerve [6, 7]. What we found in the optic nerve F-fibres was that although there was an increase in electrical threshold with the application of light, the size of fast electrotonus was unchanged, strongly suggesting that the functionality of myelin was unaltered at the same time (Fig. 6).

### Predicting changes in threshold brought about by laser light application

**Fig. 7:**
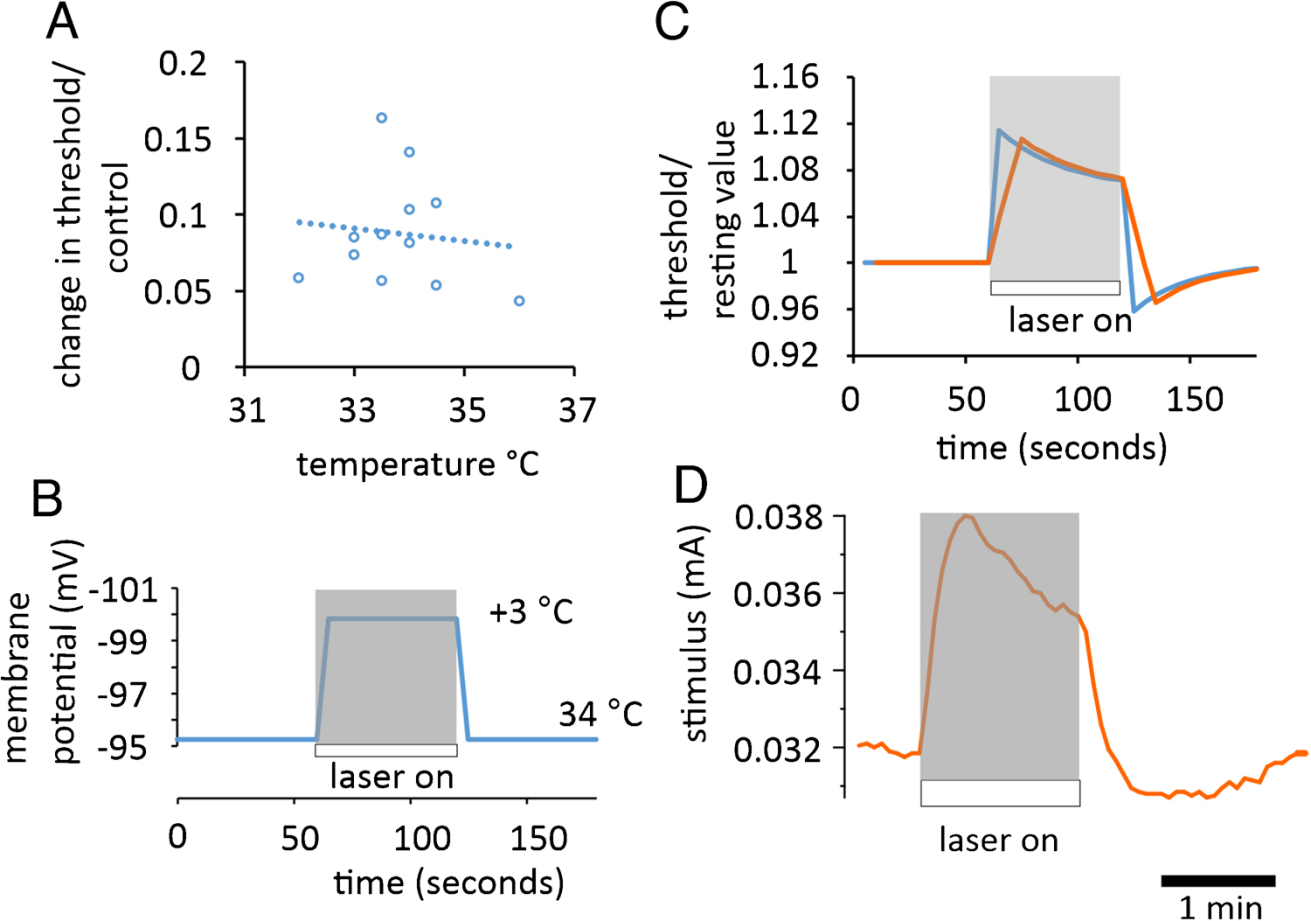
Laser light application causes changes in threshold explained by a hyperpolarization induced by local warming. **A** Application of laser light with 400 mA driving current produces a near 10% increase in threshold compared with rest (*n* = 12 nerves), apparently independent of the bath perfusion temperature (dotted line, best-fit regression). **B** The change in membrane potential changes calculated from Eq. 3 in Austerschmidt et al. [2], where the local application of IR laser light gives rise to a change in nerve temperature of 3 °C. The axonal properties were the same as those assumed previously [2], with a 40% activation of *I*_h_. C) The change in threshold brought about by the potential changes calculated in **B** modified slightly to allow the Na^+^/K^+^-ATPase to generate a gradual fade in hyperpolarization (ending with the termination of the light application at a membrane potential 1.7 mV *less* hyperpolarized than in **B**) (blue trace), with a reducing pump-current. This is consistent with the ATPase depleting the local intra-axonal [Na^+^]. In order to convert to an estimate of induced threshold change, the membrane potential changes are expressed as a fraction of the potential difference required to reach − 55 mV (taken as the threshold potential for Na^+^ channel activation), normalized with respect to the potential difference from the normal resting membrane potential value. In this scenario, the change in threshold is close to 10%, similar to experimental findings. In order to approximate the lag associated with threshold-tracking, the same data are presented following simple box-car filtering (moving average *n* = 3, orange). **D** Example threshold-tracking recording of the effect of laser light application, with a convincing after-effect on threshold, following light offset, and a nearly 19% increase in threshold during illumination (ambient bath temperature in this experiment, 34 °C). **A** and **D** Experimental data, perfusion temperature 32–36 °C

Austerschmidt et al. [2] developed an equation for calculating membrane potential that included the term *I*_EN_ (their Eq. 3). *I*_EN_ is a transmembrane current, generated by the Na^+^/K^+^ ATPase, that is not involved in returning Na^+^ ions entering via ion channels across the membrane, but rather in ejecting Na^+^ ions entering the axon electroneutrally, as a part of ion exchange or co-transport. In the Austerschmidt et al. [2] analysis, *I*_EN_ was assumed to have a *Q*_10_ and increase with temperature and in this way, the discovered temperature dependence of the axonal resting potential could be explained. The expectation, previously deduced [1, 2] that at steady-state, a 1 °C rise in ambient temperature should hyperpolarize the membrane potential up to − 2 mV, seems closely in line with what is found by applying IR laser light, where a 2–3 °C increase in local temperature, at the site of stimulation, is all that is required to explain the magnitude of changes in threshold seen in optic nerve F-fibres. Our previous work has shown that over the baseline temperature range used for the bath perfusate, a step increase in temperature should theoretically give rise to a slightly greater than linearly increasing threshold change, dependent on the baseline value, as the temperature versus threshold relation is a U shape [22]. The imposed step change in temperature thus forms part of the right-hand side of the relation. The average increase in threshold brought about by supplying the laser with 400 mA is 8.8 ± 1.0% (mean ± SEM, *n* = 22 measurements in 12 nerves) at bath temperatures between 32 and 36 °C, and with no apparent relation between the bath temperature and the effect of the laser discernible over this range. The calculated example shown in Fig. 7 involves an induced change in temperature of 3 °C and gives rise to a threshold change of 10%. However, we suggest to approximate the changes in the threshold observed, the expulsion mechanism for Na^+^ has to respond quickly and in a more sustained fashion than the influx mechanism, allowing for a local depletion of intra-axonal Na^+^, and an after-effect. We believe this scenario fully explains our findings.

## Discussion

The results presented seem consistent with a light-induced hyperpolarization resulting from a change in nerve temperature of around + 3 °C, with a 400-mA driving current to the diode. Our experiments with Li^+^ ions and bumetanide applied together suggest they depolarize the nerve by less than + 10 mV, at least initially, and this change, deduced from the altered recovery cycle, can be used to predict the reduced amplitude of the measured threshold change on light application. This is explained further below.

### Changes in trans-myelin resistance unlikely to be causing increases in threshold

As the myelin sheath, e.g. a large peripheral axon, presents an electrical resistance of around 50 MΩ, and the nodes of Ranvier will be of the order of GΩ [12], it seems safe to conclude that it is the changes in myelin resistance that will dominate any estimate of axonal input resistance measured by passing constant currents into the whole nerve. Because the input resistance was unchanged during illumination, we have ruled out an effect of laser light directly on the myelin sheath as a mechanism for increasing the threshold. As already described, the size of the peak change in threshold was reduced in the presence of Li^+^ and bumetanide, and this pharmacological effect is highly suggestive of an axonal basis for the threshold changes, rather than alterations in myelin.

Our data appear to be consistent with a previously deduced electrogenic membrane potential in optic nerve fibres that can be modified by changing temperature. The true rate of threshold increase is not always observable in our recordings, and we think this results from a software tracking rate that is too slow to faithfully follow the rate of increase, but which nevertheless follows longer-lasting increases. We suggest that the rapidity of the response implies that the Na^+^-pump must, at least partly, be anatomically present at a region of the nerve fibres that is subject to rapid warming, perhaps the node, and likewise, most of the Na^+^ influx mechanism is likely to be found under the reflective and opaque myelin sheath, therefore warming more slowly and perhaps less. There is good evidence for the expression of the Na^+^/K^+^-ATPase at both nodes of Ranvier and at internodal locations, based on a number of histological investigations, including evidence from human CNS [23, 24, 32, 34], with the probable expression of more than one isoform of the pump in neurons. As the Na^+^ pump is electrogenic, because of an asymmetry in transmembrane monovalent cation transport, its activity is associated with a hyperpolarization [27] and therefore an increase in threshold [5, 12]. Na^+^ influx mechanisms that could exhibit an appropriate temperature dependence we suggest may correspond to the equivalent of a mouse neutral amino-acid transporter (NAT) of the type reported by Gu et al. [17] in optic nerve fibres, or solute-carrier SLC38A in humans, in parallel with the bumetanide sensitive NKCC1 [1, 22]. While the solute carrier may be defined, in isolation, as electrogenic, transferring inward one unit of charge (a Na^+^ ion) with the necessary solute, if the SLC is found in a membrane permeable to K^+^ ions, the overall movement of charge will be close to zero through the simultaneous loss of an intracellular K^+^ ion.

### Nature of threshold changes caused by the application of laser light and the fade in threshold increase

Application of laser light at the site of stimulation and at bath perfusion temperatures from 32 to 35 °C caused an immediate increase in threshold, precipitated by a rapid failure of unit recruitment at the stimulating electrode. This means that small changes in temperature can prevent excitation in normal axons. The change in threshold has been described as an initial rapid effect that subsequently fades leaving an increase in threshold maintained over a minimum of 2 min [2]. We can sometimes identify a period of lowered threshold following after laser light application and suggest that the fade in threshold increase may be caused by a local depletion of intracellular Na^+^. This would be expected to reduce pumping, by reducing the saturation of the ATPase Na^+^ binding site on the inside of the membrane. The *K*_0.5_ for Na^+^ is close to 10 mM, so pump current is expected to be dependent on the intracellular Na^+^ ion concentration across the physiological range [26]. This fade is perpetuated following the termination of light application, giving rise to a period of lowered threshold, and the resting threshold subsequently takes time to return to pre-application levels. In our most stable recordings, this after-effect takes around 2 min to disappear, and we suggest this is because intra-axonal levels of Na^+^ probably remain depleted for around 2 min. This explanation is attractive because it is consistent with our findings from a paired application of laser light. Evidence for the involvement of the Na^+^ pump in the sensitivity to changing temperature is based on the block of the membrane potential effects of gross changes in perfusion temperature, by ouabain [14].

### How long does diffusion of ions take within an axon?

**Fig. 8:**
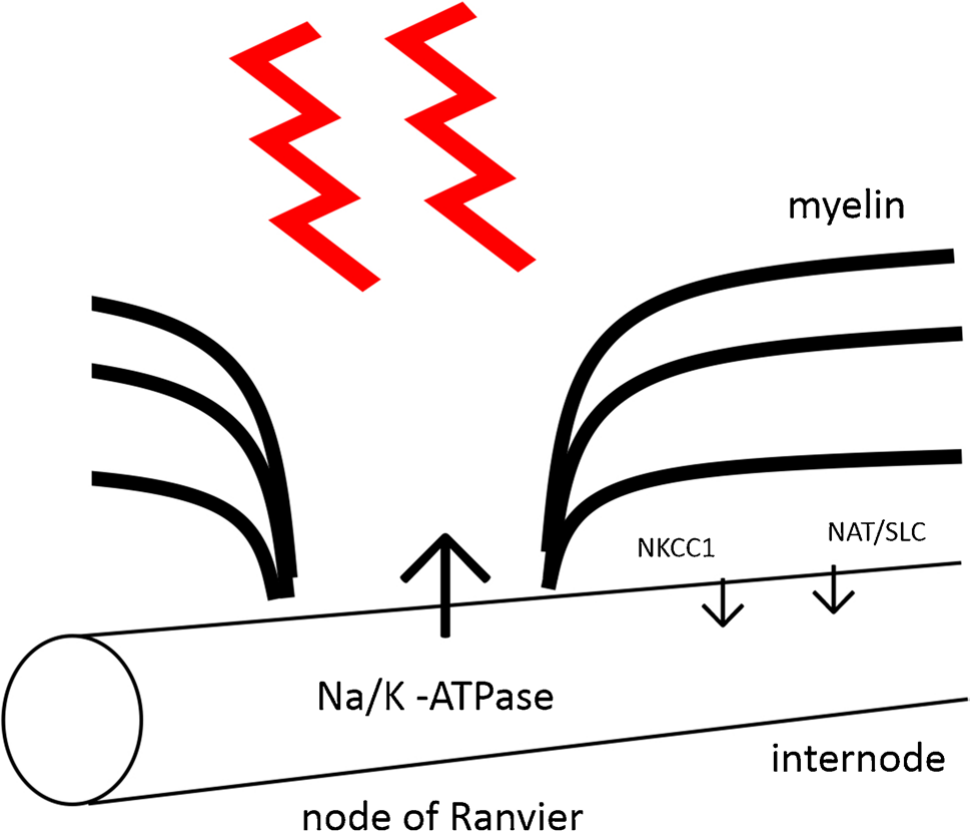
Explanation of changes in transmembrane Na^+^ movements in response to laser light illumination. Our hypothesis is that, unlike the uniform warming caused by raising the perfusion temperature, the onset of illumination rapidly and locally warms the axons, by a few single °C, but the influx mechanisms respond less than the efflux, generating a local mismatch between the Na^+^ entry and exit. This mismatch allows the ATPase to deplete the intra-axonal Na^+^ ion concentration, giving rise to a slow reduction in the electrogenic hyperpolarization over time during illumination and causing the membrane potential to drop by single mVs. In order to explain how the transport mechanisms may be differentially affected by laser light, we suggest that the pump is mainly at the node, and the hypothesized influx mechanisms including anion-cation cotransport and neutral amino acid transport are indicated as primarily internodal, under the myelin sheath. Such a situation might give rise to differential axolemma warming and perhaps allow substrate depletion in the internode. This scenario is an interpretation of the fade in threshold increase, seen in experiments such as in Figs. 2D and 3B

From our experiments with paired laser light applications, the return of the control laser-induced threshold change requires 1–2 min to complete and has a time constant of around 24 s. One possibility is that the Na^+^ replenishment route determines the rate of this process. The chemical diffusion coefficient of Na^+^ at room temperature is 1.33 *10^−5^ cm^2^ s^−1^, which means a Na^+^ ion can diffuse in a single direction across 100 microns of water in about 4 s [31]. Given the small size of optic nerve axons, it is unlikely that any intra-axonal diffusion has to occur across 100 microns, and it therefore seems safe to conclude that the long time constants we have measured must arise from differences in the rate of influx and efflux mediated by transmembrane Na^+^ transport systems integrated over time, that slowly changes the concentration of Na^+^ within the axon. Further, under normal circumstances where the whole nerve is affected by changes in temperature, our expectation is that changes in influx and efflux rates will balance exactly and that this phenomenon arises because the transport mechanisms are affected differentially by the laser (Fig. 8). We also conclude the intra-axonal depletion of Na^+^ ions must likely be corrected by influx, rather than by ion redistribution.

The equations from Austerschmidt et al. [1, 2] that calculate transmembrane membrane voltage changes associated with altered temperature make the assumption that intracellular Na^+^ does not change when the temperature does. This assumption may well be correct for changes in temperature that affect the whole nerve. However, a simple explanation for the slow changes in threshold we measure during light application (and also following light application) may include depletion and subsequently replenishment of intracellular [Na^+^].

### What underlies the reduced laser-induced threshold change in the presence of lithium and bumetanide?

In the presence of extracellular Li^+^ and bumetanide, the effect of the laser on threshold is not abolished, but reduced, before ongoing gross changes in threshold with continued perfusion make it difficult to follow the effects of illumination. One possibility that may explain these findings is that there is a reduced steady-state intra-axonal [Na^+^] and a proportional reduction in the increased Na^+^ entry with illumination. If this were true, it might be consistent with most of the resting Na^+^ influx into the axon being dependent on Li^+^ blockable solute transport. An alternative explanation, however, is based on the hypothesis of a build-up of Li^+^ intracellularly that reduces the efficiency of the Na^+^ pump, directly limiting any light-enhanced hyperpolarization. It may not yet be possible to rule out the direct pump-inhibition scenario. The combination of Li^+^ and bumetanide does appear to cause a depolarization of the membrane potential, because refractoriness increases with simultaneous large increases in threshold following prolonged exposure, where the latter is consistent with an increase in resting Na^+^ channel inactivation. A loss of Na^+^ channel function alone seems an inadequate explanation for the combination of effects we report, because it would not account for the increase in refractoriness—that requires a depolarization.

The degree to which the laser-induced threshold increase is diminished by the combination of Li^+^ and bumetanide can be predicted from the estimated loss of steady-state electroneutral Na^+^ flux. In the experiment shown in Fig. 4, the changes in the characteristics of the recovery cycle given in panel B imply a change in membrane potential of around + 6 mV, in order to be consistent with the data published in Austerschmidt et al. [1]. Also, our estimate of the relationship between temperature and membrane potential given in the same 2020 publication means that the equivalent change in temperature for a + 6 mV change in membrane potential lies between − 3 and − 4 °C. Therefore, the estimated fall in electroneutral flux is given by:

1 $${X}_{t}/{3}^{(3/10)}or{X}_{t}/{3}^{(4/10)}$$

assuming a *Q*_10_ of 3. *X*_t_ is the number of moles/s of Na^+^ entering per unit length of axon at a given temperature. In this example, the electroneutral flux and hence *I*_EN_, the Na^+^-pump mediated return current, must fall to between 72 and 64% of the flux before Li^+^ and bumetanide application, and this would be consistent with a similar proportional fall in the laser-mediated effect. Overall, our data on the laser are consistent with a block of the effect of light, but on average, the laser effect is blocked more than predicted. An explanation for this is that the Li^+^ and bumetanide not only reduce Na^+^ ion flux, but also reduce the intra-axonal Na^+^ ion concentration, and this would be expected to reduce the light-induced hyperpolarization in its own right.

### How might threshold changes brought about by changing temperature in the optic nerve be related to MS symptoms?

The changes in threshold we have measured with the laser light application and the deduced changes in membrane potential are rather small (the latter within the single mV range). The increases in membrane potential over a few degrees centigrade give rise to changes in the electrical threshold of 10% (or a bit more) of the current used to stimulate. However, this is indisputably large enough to cause excitation failure in some normal axons at the stimulating electrode and to suppress the amplitude of evoked compound action potentials while irradiation continues. It is for these reasons that we suggest our findings have a probable relevance to temperature-dependent impulse conduction failure in diseased axons. In multiple sclerosis (MS), axons can be compromised by a reduced safety factor for impulse propagation, and MS is a notoriously temperature-sensitive disease where conduction failure is understood to increase with warming, e.g. [11].

In MS, central white-matter axons are understood to be demyelinated by local inflammatory activity. Under these circumstances, widened nodes or segmental demyelination, even with changed Na^+^ channel expression and redistribution [10], must be associated with raised electrical capacitance at sites of impulse initiation and thus requiring a greater depolarizing local circuit current for recruitment. Raised temperature would thus be a burden at these sites by increasing the resting potential, moving the axolemma further from the recruitment threshold for Na^+^ channels, and by shortening the action potential duration, limiting action currents [10, 11, 28]. The intra-neuronal Na^+^ ion concentration must be maintained normally within fairly tight limits, inevitably linked to levels of ATP production and mitochondrial sufficiency. Na^+^ ion accumulation has recently appeared as a marker for neurodegeneration in Alzheimer’s disease [25] and for MS [9, 21]. Because the electroneutral movements of Na^+^ must be substantially bigger than those occurring through open Na^+^ channels, they may play a part in long-term cellular energy deficits in the brain and therefore in neurodegeneration.

Our observations on glutamine loading of the optic nerve, although preliminary, suggest that Na^+^ movement may be importantly related to amino-acid uptake on an axonal NAT/SLC, and our threshold measurements could represent a method of exploring the phenomenon. In the context of central demyelinating disease (and perhaps a release of substrates from the ensheathing cell), loss of glia would cause a fall in periaxonal substrate concentration and potentially stress the axons by locally starving them and conceivably suppressing local mitochondrial ATP production (because, for example, glutamine will feed into Krebs cycle via alpha ketoglutarate). Such a neuronal/glial interactive unit has already been hypothesized to involve lactate and monocarboxylate transporters and has been summarized elsewhere e.g. [30].

## Data Availability

The datasets used during the current study are available from the corresponding author on reasonable request.
